# Visual expertise for aerial- and ground-views of houses: No evidence for mental rotation, but experts were more diligent than novices

**DOI:** 10.1177/03010066251378983

**Published:** 2025-10-07

**Authors:** Emil Skog, Andrew J. Schofield, Timothy S. Meese

**Affiliations:** 1 School of Psychology, College of Health and Life Sciences, 1722 Aston University, Birmingham, UK; 2 Aston Laboratory for Immersive Virtual Environments, College of Health and Life Sciences, 1722 Aston University, Birmingham, UK; 3 Department of Health, Learning and Technology, Luleå University of Technology, Luleå, Sweden

**Keywords:** mental rotation, viewpoint invariance, feature identification, object recognition, experts, unusual viewpoints

## Abstract

Ordnance Survey (OS) remote sensing surveyors have extensive experience with aerial views of scenes and objects. Building on our previous work with this group, we investigated whether their expertise influenced performance on a same/different object recognition task involving houses. In an online study, these stimuli were shown from both familiar ground-level viewpoints and from what is for most people, unfamiliar aerial viewpoints. OS experts and novices compared achromatic, disparity-free images with aerial perspectives rotated around the clock against canonical ground-views; we measured response times (RTs) and sensitivities (*d’*). In two ‘grounding’ tasks using rotated letters, we found conventional outcomes for both groups, validating the online approach. Experiment 1 (non-matching letters) yielded ceiling-level performance with no signs of mental rotation, consistent with a feature-based recognition strategy. In Experiment 2 (mirror reversed letters), both groups showed orientation-dependent performance, but experts exhibited a speed-accuracy trade-off, responding more cautiously than novices. In the main house task (Experiment 3), we found (a) the same speed-accuracy trade-off observed in Experiment 2, (b) substantially longer RTs overall, and (c) no evidence for mental rotation in either group, mirroring Experiment 1. Contrary to our earlier findings on aerial depth perception, expertise in remote sensing did not yield a distinctive recognition strategy for the experiments here. However, experts displayed more diligent tactics in Experiments 2 and 3. We suggest that all participants in Experiment 3 engaged in cognitively challenging feature comparisons across viewpoints, presumably supported by volumetric or surface-connected prototypes of houses as the basis for feature comparisons.

## Introduction

### Viewpoint Invariance and Mental Rotation

Objects seen from unfamiliar viewpoints are normally more difficult to recognise (e.g., Biederman & Gerhardstein, 1993; Lawson, 1999; Newell & Findlay, 1997). Nonetheless, *viewpoint invariance* – the ability to recognise an object from multiple viewpoints (e.g., Humphreys & Riddoch, 1984) – is often treated as a general property of human object recognition (Marr, 1982), albeit stimulus-dependent (Blais et al., 2009) and computationally challenging (Hummel, 2000; Ratan Murty & Arun, 2015; Tarr & Cheng, 2003). Evidence for viewpoint invariance appears as early as 3-months of age (Kraebel & Gerhardstein, 2006), though object recognition continues to develop beyond infancy (e.g., Jüttner et al., 2013, 2014; Rentschler et al., 2004). Development studies have shown viewpoint invariance in both chicks (Wood & Wood, 2016) and humans – behaviourally (Rentschler et al., 2004) and in the lateral occipital cortex (Nishimura et al., 2015). Moreover, viewpoint invariance can be enhanced by domain-specific training (e.g., Ashworth & Dror, 2000; Tarr & Cheng, 2003), and is central to training in machine vision systems (e.g., Ruan et al., 2023; Salari et al., 2022).

A related visuo-cognitive skill is *mental rotation* (though see Hayward et al., 2006; Jolicoeur et al., 1998). This is the ability to rotate 2D or 3D object representations in the ‘mind's eye’ (Shepard & Metzler, 1971), or through an operationally equivalent mental operation (Kay et al., 2024; Pounder et al., 2022; Zhao et al., 2022). In a typical experiment, response times (RTs) for same/different judgements increase with the angular disparity between objects (Shepard & Metzler, 1971). Mental rotation has been studied using brain imaging techniques (e.g., see Bode et al., 2007; Gauthier et al., 2002; Kosslyn et al., 1998; Schendan & Stern, 2008; Zacks, 2008; Zacks & Michelon, 2005) and its developmental trajectory has been measured across infancy and childhood (Estes, 1998; Frick et al., 2013; Kail et al., 1980). Training has been shown to affect mental rotation characteristics and strategies (Bethell-Fox & Shepard, 1988; Jost & Jansen, 2022; Kail & Park, 1990; Koriat & Norman, 1985; Leone et al., 1993; Meneghetti et al., 2017; Moreau, 2013), and expertise – particularly motor or embodied expertise – can modulate performance (Habacha et al., 2022; Jansen & Lehmann, 2013; Pietsch & Jansen 2012; Voyer & Jansen, 2017). Although this visuo-cognitive ability remains largely intact in older adults, mental rotation speed declines with age (Band & Kok, 2000; Dror & Kosslyn, 1994). In sum, both viewpoint invariance and mental rotation are malleable visuo-cognitive capacities that depend on specific neural circuits and are shaped by visual experience and expertise.

### Remote Sensing Surveying, Object Recognition and the Motivation for Our Study

As part of an ongoing collaboration with Ordnance Survey (OS) – the national mapping agency for Great Britain – we sought to better understand the visuo-cognitive processes used by OS experts when interpreting aerial scenes. Our previous studies examined depth perception in aerial images (Skog et al., 2024) and gist perception for scenes viewed from ground level and above (Skog et al., 2025). In the current study, we extended this work to object recognition by investigating whether expertise in aerial interpretation influences behaviour on same/different decisions involving objects (in this case, houses) viewed from familiar ground-level and unusual/unfamiliar (for most people) aerial viewpoints. We did this by comparing task behaviours of OS remote sensing surveyors with novices from the general population.

Remote sensing surveyors are trained to identify and classify landscape features – including houses – from aerial perspectives, as required by their photogrammetry profession. Several previous studies have identified benefits of experience with aerial images. For example, in categorisation tasks, Borders et al. (2020) found improvements in speed and accuracy; Lloyd et al. (2002) reported enhanced speed and confidence; and Skog et al. (2025) observed better accuracy. Other studies have shown enhanced scan path efficiency (Lansdale et al., 2010), improved recognition memory (Šikl et al., 2019), and better use of binocular stereopsis (Skog et al., 2024). These benefits emerged either through training (Borders et al., 2020; Lloyd et al., 2002) or in group comparisons between visual experts and novices (Lansdale et al., 2010; Šikl et al., 2019; Skog et al., 2024, 2025).

However, to our knowledge, no previous study has examined the ability of expert remote sensing surveyors to match objects across ground and aerial viewpoints. Although this type of task is not routine for OS experts, some have experience in matching aerial photographs taken from high-flying aircraft with oblique-angled photographs from lower-flying drones (OS personal communication). Other research suggests that abilities such as mental rotation and spatial perspective taking might play a role in this type of task. For example, Lokka and Çöltekin (2020) found links between mental rotation and the ability to switch between ground and aerial route plotting, and Huang and Voyer (2017) reported associations between the cognitive skills needed for aerial views and maps, perspective taking, and relating aerial views with 2D cross-sections. Neuroimaging research on mental rotation has found differences in specific brain activity (in dorsolateral prefrontal cortex) for participants with STEM backgrounds and those without, implying a pivotal role for spatial reasoning in this task (da Silva Soares Jr et al., 2025) – a key skill for remote sensing surveying.

Finally, in a classification image (CI) study of aerial views of hedges and ditches, Skog et al. (2024) found striking differences between OS experts and novices. Experts prioritised binocular disparity cues over luminance cues whereas, surprisingly, novices had greater CI luminance amplitudes than the experts. Not only did this show different hedge/ditch identification strategies across groups, but also ruled out motivational factors as an explanation. In fact, novices were more sensitive (higher *d*′) than experts for implicit lighting direction cues, even though neither group was aware of using this cue.

Taken together, these findings led us to ask whether aerial expertise might affect performance in our object recognition task involving comparisons across ground and aerial views. Several possibilities arise. For instance, experts and novices might differ in mental rotation speeds or might rely in different strategies for the task. OS surveyors might, for example, be especially adept at mentally rotating aerially viewed objects as part of a general recognition strategy – an approach potentially less accessible to novices. More generally though, it is currently unknown whether observers (experts or not) use mental rotation when comparing complex real-world stimuli such as photographs of houses.

In sum, the primary goal of our study was to investigate the visuo-cognitive performance and recognition behaviours of aerial image experts compared to novices. This positioned our study as an open investigation of visual expertise in same/different object recognition in the context of aerial viewpoints, rather than a targeted test of mental rotation ability of visual experts. Nonetheless, we are unaware of any prior study that has investigated potential 3D mental rotation for houses including unusual (aerial) views in any participant group. While Bode et al. (2007) reported mental rotation effects for house drawings (amongst other stimuli), they did not include aerial viewpoints. In their study, houses were judged to be more difficult to rotate than tools, hands, or 2D figures, but less difficult than Shepard and Metzler's (1971) 3D ‘brick’ objects.

We did not formulate specific hypotheses for our experiments, but we interpret our results in the light of existing work on mental rotation and object recognition.

### Real World Issues and Experimental Design

Visual object constancy is relatively easy to achieve when all parts of an object remain visible across different viewpoints. For example, a 45° horizontal rotation can be arranged to avoid occlusion or disocclusion of parts (Biederman & Gerhardstein, 1993; Lawson, 1999). However, switches from ground to aerial views often change which object features are visible – or which features are emphasised at least. For example, a typical ground-view of a house emphasises the elevations, while the roof is compressed or substantially occluded in the retinal image. In contrast, an aerial view emphasises the roof with the elevations mostly absent from the 2D image. Clearly, feature-matching across disparate viewpoints presents a significant challenge for any visual system.

For most people, aerial perspectives are relatively unfamiliar, compounding recognition difficulty (Center et al., 2022; Edelman & Bülthoff, 1992; Newell et al., 2001; Tarr et al., 1998). In general, recognition is most efficient for canonical views – those that are ‘best’ or ‘preferred’ from an observer's perspective (e.g., Palmer et al., 1981). Rotating an object away from its canonical orientation usually impairs recognition. For ground views, canonical orientation is aligned with the gravitational frame (Asch & Witkin, 1948; Dyde, Jenkin & Harris, 2006; Loschky et al., 2015; Mittelstaedt, 1983). In aerial views, however, the gravitational frame is fronto-parallel to the observer and façades can face any direction in the 2D image plane. In other words, there is no obvious 2D canonical orientation for aerial images (Loschky et al., 2015). On the other hand, our stimuli often included contextual cues (e.g., surrounding landscape, though this varied across our stimuli), which can aid recognition under non-canonical conditions (Kallmayer et al., 2023).

Thus, the primary difference between expert and novice observers in this context may be the experts’ greater familiarity with atypical viewpoints, potentially facilitating object recognition.

In our study, ground-view photographs were always taken facing the façades of houses, and their orientation was held constant. By contrast, we systematically varied the 2D orientation of aerial images so that façades faced in several directions around the clock. In principle, to determine whether two images depict the same house, participants might mentally rotate the aerial image to align it with the façade of the ground-view. Under this strategy, a single 3D rotation from aerial to ground view is nominally constant across trials, while the 2D rotation within the aerial image plane is the variable under experimental control. Whether this mental rotation occurs as two steps (2D aerial, followed by 3D from aerial to ground) or one (a direct 3D rotation from one image to the other) we would expect RTs to increase with orientation difference (Cooper & Shepard, 1973; Parsons, 1987; Shepard & Metzler, 1971).

An alternative strategy is feature-based identification (White, 1980) where salient features (e.g., window placements, roof shape) are compared across views without the need for mental rotation (Smith & Dror, 2001). Under this approach, RTs should be largely unaffected by orientation differences (we consider this further, below). Nonetheless, regardless of visuo-cognitive strategy, we reasoned that differences in experiences with aerial images could lead to group differences in results.

Given the plausibility of at least two strategies in our house comparison task (we consider a third in the *General discussion*), we conducted two additional experiments using familiar letter stimuli to ‘ground’ the main experiment. In a basic version of the task, participants judged whether letter pairs were the same or different. This can be done by simple feature comparisons (e.g., a ‘P’ has a closed curve absent in an ‘L’), without requiring mental rotation. Consequently, orientation differences across stimulus pairs should have little or no effect, RTs should be fast, and accuracy high (Corballis et al., 1978; Milivojevic et al., 2011; Takano, 1989; White, 1980).

In contrast, this strategy cannot be used when the same letters are compared but one might be a mirror reversal of the other. In this case, the features are the same and so mental rotation is required to determine whether the letters are mirror reversed, with RTs increasing with their orientation difference (e.g., Hamm et al., 2004; Koriat & Norman, 1985; Milivojevic et al, 2011; Núñez-Peña & Aznar-Casanonva, 2009; Takano, 1989; White, 1980).

These two letter-based tasks were intended to anchor interpretation of the main experiment. For example, if group differences emerged in Experiment 3 (houses), it would be important to assess whether similar group differences occurred for tasks unrelated to aerial expertise. If so, then group effects could not be attributed to domain-specific training. Furthermore, because the letter tasks have well-established behavioural outcomes (see above), they also served to validate the online format of our study – perceptual comparisons such as those here are typically conducted under laboratory controlled conditions.

## Methods

### Participants

Twelve expert participants were recruited from OS (7 female; mean age: 40 years [SD: 10]; mean experience with remote sensing surveying = 10 years [SD: 5], range: 1–25 years). Thirteen novice participants were recruited from Prolific Academic (www.prolific.com), but one was excluded for failing an attention check question (see *Procedure* below) (8 female; mean age: 38 years, SD: 12). The novices had an average of 440 (SD: 459) approved participations in other studies and surveys on Prolific (one of these had 1,597 approved participations; only three had fewer than 62). All participants were fluent or native speakers of English and based in the UK or Ireland. The total number of participants was constrained by the number of expert remote sensing surveyors prepared to volunteer, and these were matched in number by novices. All participants completed all three experiments in immediate succession in a counterbalanced order (see below). The experiments took around 30 min to complete, and participants were compensated with £5.

The project was conducted with ethical approval by Aston University's College of Health and Life Sciences Ethical Review committee (#1843) and all participants gave informed consent via a button press. All experts indicated they had significant experience with aerial images and all novices indicated they had not.

### Equipment and Software

The experiment was created using PsychoPy (version 2021.2.0; Peirce et al., 2019) and JavaScript to run on the online experiment delivery platform Pavlovia (Pavlovia.org). Participants used their own desktop computers to run the experiment in their web-browser. The experiment was accessed via a hyperlink provided by email to the experts and by Prolific for the novices. The computer, monitor, mouse and keyboard, viewing distance, and testing environment were not controlled. PsychoPy handled stimulus timings and recorded RTs, which are known to be reliable across different operating systems and web browsers (Bridges et al., 2020).

### Stimuli

Same/different judgements were made across stimulus pairs in all experiments and the instructions and stimuli were always achromatic (a mid-grey background and black text including letter stimuli and grey-level photographs of houses). Images were monoscopic, with the same image presented to both eyes.

#### Experiment 1: Letter Rotations

In Experiment 1, the stimuli were six different capital letter pairs: ‘F’, ‘G’, ‘J’, ‘L’, ‘P’, and ‘R’. (The letters were selected to have different main features using consonants for consistency.) On each trial, both letters were subject to rotations in ±45° steps to give eight randomly selected base orientations (0°, ± 45°, ± 90°, ± 135°, and 180°) and five systematic orientation differences across the pairs (0°, 45°, 90°, 135°, and 180°). For each (5 × 6) combination of orientation differences and letters, the second letter was either the same letter of the alphabet or a different letter, randomly selected from the other five. This gave a total of 60 trials per observer.

**Figure 1.**
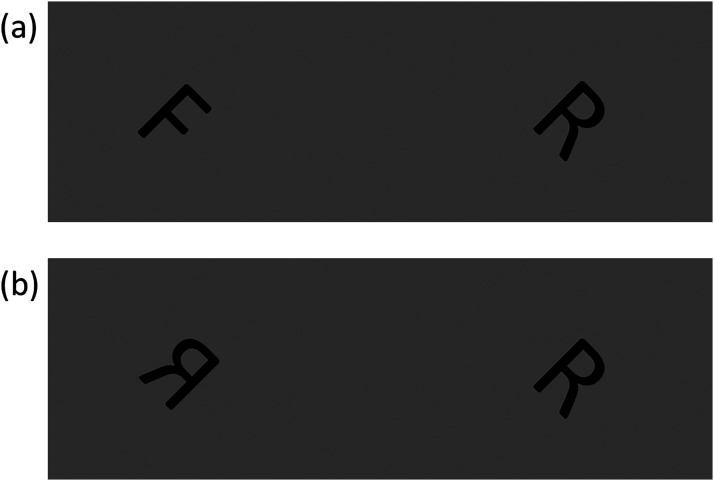
Example of a letter pairs used in the two letter experiments. In both examples the absolute orientation is 45 deg, the orientation difference is 0 deg, and the letters are for the ‘different’ condition. (a) Example from Experiment 1 where the letters are different letters of the alphabet. (b) Example from Experiment 2 where the letters are mirror reversed.

The letters were presented in ‘Calibri’ font with a height of 9.5% of that of the participant's display. The letter width was determined by the font and was less than the height. Letters were adjacent on the horizontal centreline of the display with a centre-to-centre spacing of 41% of the display height between them. An example letter pair is shown in Figure 1(a).

#### Experiment 2: Mirror Reversals of Letters

Experiment 2 used the same letters, orientation conditions and number of trials as in Experiment 1 but replaced the ‘different’ letter of the alphabet pairs with a mirror-reversal of the same letter (about its vertical midline). The mirror-reversal was applied to the letter on the left or right of the display with equal probability. When the letter pairs were the same, either both or neither of the letters was mirror reversed with equal probability. An example letter pair is shown in Figure 1(b).

#### Experiment 3: Houses

Ground-view images of houses^1^ were collected by photography in a suburban district of Birmingham, UK, using a 48-megapixel camera. Photographs were taken roughly face-on (see below) to capture all the details of the façade and assigned a nominal orientation of 0°. Aerial images were sourced from the OS and cropped to isolate individual houses. These included images of the same houses from the ground views identified by house number and street name. In some of these cases, vegetation and movable objects such as cars changed across viewpoints, meaning occasional ‘non-house’ features were inconsistent across images. Ground-view and aerial photographs were captured in October 2022 and July 2022, respectively.

One-hundred ground-view houses were chosen to create ‘same’ pair stimuli by pairing with aerial-views of the same houses. A further set of 100 ground-view houses were chosen to create ‘different’ pair stimuli by associating each one with an aerial image of another house which differed in either one or two of the following features (and was similar in the others): (1) house shape outline, (2) roof shape (e.g., gabled or hipped roof), or (3) façade and roof features (e.g., bay windows, chimneys, or dormer windows). The houses in each pair were always similar in size and sourced from the same street or a similar-looking street, nearby. Façades were visible in all ground and aerial images of the houses such that diagnostic image information pertinent to matching the houses across views was available in every pair. In several aerial images, the façade detail was severely limited but, we reasoned, sufficient for the task. In some aerial images, the back of the house was cropped out of the image, but no diagnostic information was lost because the house backs were never visible in the ground-view images.

**Figure 2.**
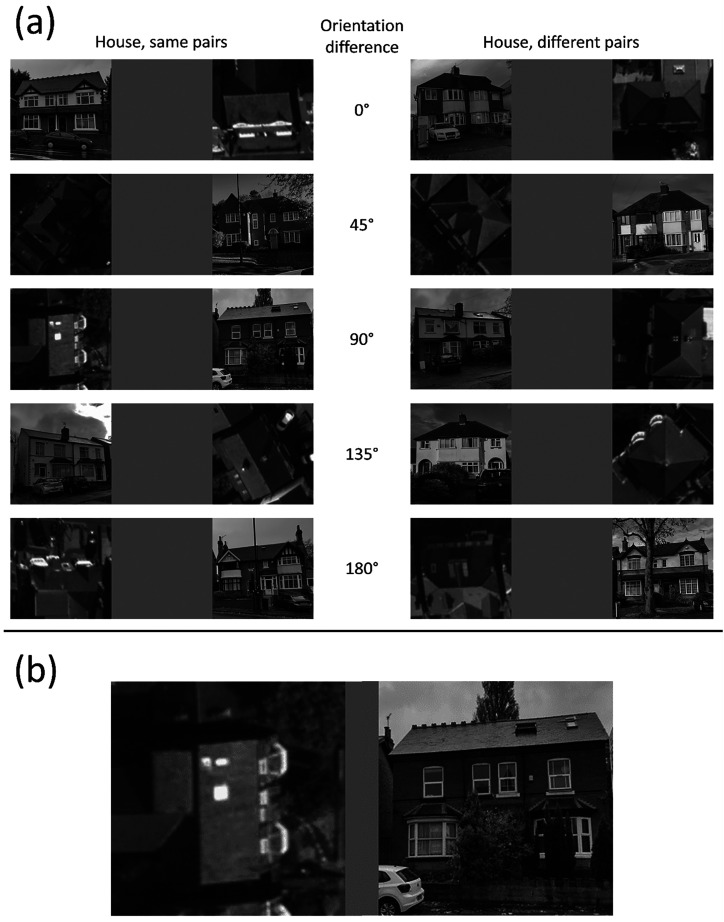
Example stimuli used in the house experiment for both the ‘same’ and ‘different’ conditions. Orientation difference conditions were determined by the orientation of the aerial image. Panel (a): From top to bottom, unsigned examples are shown from 0° to 180°. The ground views are face-on with a tolerance of ±0.5 bin width of orientation difference (±22.5°) determined by eye. The aerial views had a tolerance of ±0.5 bin width from their nominal orientation determined by eye. Panel (b) shows an enlarged version of ‘same’ pairs, 90°. aerial images: *^©^ Crown Copyright and Database Rights 2025 OS*, used with permission.

The orientation of the ground-view house images was not an independent variable. For the aerial views of houses, orientation varied around the clock in the native images which were assigned to nominal categories in 45° steps, where 0° was for façades facing down in the 2D image plane (see Figure 2). Across categories, the mean deviation from the nominal orientation was 4.0°, and the mean SD of the distributions was 10.7°. House images varied in several parameters including street, general appearance, size, shading, and sunlight direction. As far as possible, these variations were evenly distributed across the orientation difference conditions based on casual observation. Within the 200 stimulus images, there were 20 image-pairs for each of the five nominal orientation differences for each of the ‘same’ and ‘different’ categories (i.e., a total of 100 ‘same’ house pairs, and 100 ‘different’ house pairs).

Each house image was cropped to a square, converted to greyscale, resized to 300 × 300-pixels using bicubic interpolation, and stored in PNG format. Images in each pair were placed next to each other with a 300-pixel wide space between them giving an overall width of 900-pixel (see Figure 2). The left/right order of the images was first split evenly across image-pairs, and the trial order of these was randomised independently for each participant. Each participant saw the same set of house-pairs, and no house was repeated across pairs in the set.

During the experiment, the images were scaled using linear interpolation to a height of 30% of the participant's display keeping the aspect ratio intact. On a typical laptop screen with a height of 19 cm, this produced image sizes of 5.68° square at a comfortable viewing distance of 57 cm, though no specific instructions were given about viewing distance. Ground-view images had a spatial resolution greater than 300 pixels prior to resizing, but aerial images were lower in resolution (mean square pixel area: 122, *SD*: 25), limited by the aerial photographs once cropped to contain individual houses. This meant that aerial-view houses had a consistently grainier appearance than the ground-view houses (Figure 2).

After completing the experiment, we found that several of the nominally face-on photographs taken at street level were somewhat oblique owing to the combination of the photographic lens and spatial distance (street width) available. To address this, we judged by eye which of these images produced ground-based object rotations from the photographic viewpoint of more than ±0.5 of the step size in the orientation difference conditions (i.e., ± 22.5°). Using this procedure, we identified 18 (out of 100) images amongst the ‘same’ pairs and 13 (out of 100) images in the ‘different’ pairs. These outlying image pairs were removed from the analyses in the remainder of this paper resulting in three rather unimportant changes (identified in *Results and discussion*) to thirty cells of the ANOVA (Table A1 in Appendix A).

#### Houses and Letters

In general, houses and letters are both highly familiar stimulus sets for most people. However, the vertical ground views of our house stimuli contained task-specific details that broke this familiarity (i.e., decisions needed to be made involving details specific to novel houses), but this was not generally true for vertical letters (unless they were reversed). This can be important in studies of this kind (Koriat & Norman, 1985) and so in an attempt to resolve this asymmetry across stimulus types, we always randomised the orientation of the base letter (from the available set) against which comparisons were to be made, thereby decreasing familiarity for the letters (Koriat & Norman, 1985).

### Procedure

In all experiments, instructions stated that the task would be a ‘same or different’ judgement where the ‘s’ and ‘d’ keys would be used for ‘same’ and ‘different’ responses, respectively. Participants were instructed to respond accurately and quickly. For each experiment, six exemplars and supporting text were presented to illustrate the meanings of ‘same’ and ‘different’ (three exemplars each). Participants then performed five, 10 and six practise trials in Experiments 1–3, respectively, for stimuli that were not repeated in the experimental trials. Throughout Experiment 1, text was placed at the top of the screen to remind participants to respond according to: ‘“S” key: Same letter, “D” key: Different letters’. For Experiment 2, this read: ‘“S” key: Looks the same, “D” key: Mirrored differently’. In the practice trials of Experiment 2, visual feedback was provided to indicate correctness of response to reinforce that ‘different’ in this task referred to a mirror reversal across the pair. This was done to address our finding in pilot work that some participants appeared not to understand the task requirements in this experiment. For Experiment 3, the text at the top of the screen read: ‘“S” key: Same house, “D” key: Different houses’.

Trials started with a 1500 ms interstimulus interval of a blank screen, followed by unlimited presentation time of the stimulus (i.e., the stimulus was not extinguished until the participant responded). Each response initiated the next trial, and the stimulus order was randomised. One attention check question was presented at the end of the 60 trials in Experiments 1 and 2. In this, participants were asked to identify text stating ‘red’, ‘green’ or ‘blue’ with the ‘r’, ‘g’ or ‘b’ buttons. In Experiment 3, there were four attention check questions, one after every 50 trials. Following the final attention check question in each experiment, the next block started with instructions for a new experiment, or the session terminated as appropriate. The purpose of the attention checks was to assess whether participants were attending to the display screen in their online environments. As mentioned above in *Participants*, only one participant was rejected for failing this test (i.e., making an incorrect response in the test).

### Order of Experiments

The three experiments were performed by each participant in a block design at a single sitting. The two-letter experiments were always performed contiguously. In each experimental group, six participants started with the house experiment followed by the letter experiments, and vice versa for the other six participants. The order of the letter experiments was also counterbalanced so that half of the participants in each group and block combination above did Experiment 1 before Experiment 2, and vice versa for the other participants.

## Results and Discussion

### Data Preparation and Analysis

Trials where the RT was more than two standard deviations from the mean for the respective participant were removed from the analyses. This mainly resulted in discarding very slow trials (2.8%, 3.2%, and 2.1% of trials from Experiments 1–3, respectively). Only correct responses were used in the RT analyses.

The accuracy results were converted to the sensitivity measure *d*’ (Green & Swets, 1966) to avoid potential contamination by response bias towards ‘same’ or ‘different’. To do this, ‘same’ and ‘different’ images and responses were classified as hits ('same’-'same’), misses ('same’- 'different’), false alarms (‘different’- 'same’), and correct rejections (‘different’-'different’).^2^ This necessarily combined information across ‘same’ and ‘different’ trials for our sensitivity measure. As our sample sizes were quite small (there were 20 trials per condition for each observer before removals) we advise applying caution over the precise values of these estimates (e.g., Hautus, 1995). We also assessed bias in each of our experiments.

**Figure 3.**
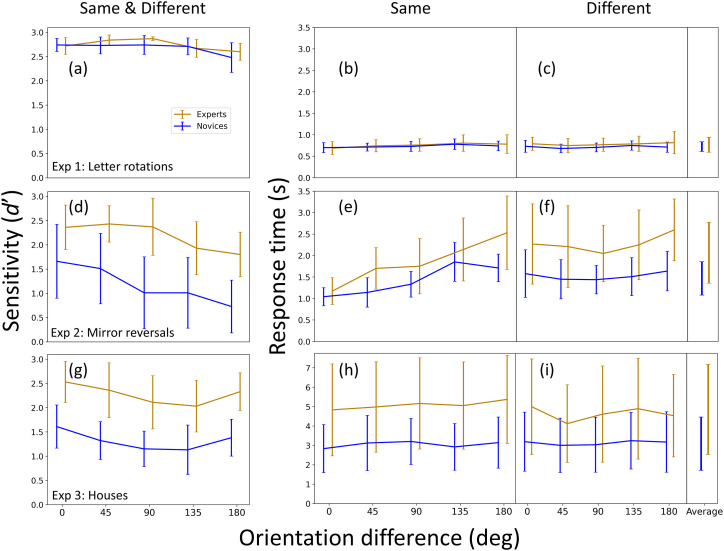
Results for all three experiments (Experiments 1–3, in rows 1–3, respectively). The left column shows accuracy (*d*’, sensitivity), and is derived from the ‘same’ and ‘different’ conditions (see text for details). The other two columns are response times (RT) in seconds (s) for ‘same’ responses (middle column) and ‘different’ responses (right column). The ‘average’ entries (far right) are the mean RT results across orientation difference conditions. Note the different Y-axes scales for RTs across the two letter experiments (Experiments 1 and 2) compared to the house experiment (Experiment 3). The RT error bars within each panel are ± within observer standard deviations (SD) across trials for each orientation difference and averaged across participants. (These are pooled in the ‘average’ column on the far right.) The error bars for the sensitivity measures are 95% confidence intervals across participants.

Frequentist statistical analyses (analysis of variance [ANOVA]) were calculated using Jamovi (version 1.6.23.0). For each of the three experiments we performed a mixed 2 (Group: novice, expert) × 5 (Orientation difference: 0°, 45°, 90°, 135°, 180°) repeated measures ANOVA with *d*’ as the dependent variable (the lefthand column in Figure 3). We also performed a mixed repeated measures ANOVA, with an additional factor (Trial type: ‘same’/‘different’) and RT as the dependent variable (the middle- and right-hand columns in Figure 3). The details of these analyses are reported in Table A1 of Appendix A. For clarity of presentation, we typically report only the outcome (i.e., significance and *p* values) of these analyses in the main body text. The details of further post-hoc statistical analyses (e.g., across experiments or with one of the factors removed) are reported within the body text.

For the purpose of discussing null effects, Bayesian factors were also calculated using JASP version 0.17.3.0 with JASP's default Bayesian priors and are reported in Appendix A as appropriate. However, owing to the small sample size (determined by the availability of OS participants) we acknowledge that our statistical analysis might have misrepresented the underlying effects.

As stated in Appendix A, Greenhouse-Geisser correction was performed as appropriate following a positive sphericity tests. Furthermore, analyses of RTs were always based on correct responses, but in Experiment 2, some participants (one expert and five novices) provided no correct responses in at least one image category (e.g., ‘same’ image, 45° orientation difference). Such image categories consisted of six trials each. To avoid excluding participants on this basis, 13 out of 240 cells were filled with the average of the other available participants in the same group and condition.

Finally, although we describe and organise our results in terms of three different experiments, we remind the reader that these were performed in a single sitting and counterbalanced across participants as outlined at the end of the Methods section. We present the two letter experiments first since these are needed to ground our interpretation of the third, main experiment, on houses.

### Experiment 1: Different Letters

The results for Experiment 1 are shown in the top row of Figure 3. Both groups completed the simple letter rotation task with almost perfect accuracies (Figure 3(a)) and RTs of around 700–750 ms (Figure 3(b) and (c)). ANOVA revealed no accuracy effect for group, orientation difference, or interaction between these factors, confirmed by the Bayes factors in Table A1. For RTs, there were no significant effects for group, orientation difference or trial-type ('same’/'different’), nor any significant interactions between these factors, with the exception of orientation difference and trial-type (*p* = .043). However, the Bayes factor favoured the null hypothesis (BF_10_ = 0.61), leading us to assign no theoretical significance to this marginal result (Table A1 in the Appendix). (Note that Shepard & Metzler (1971) separated ‘same’ and ‘different’ responses for their RT analysis and it has been common place to do this ever since. Often, only the ‘same’ trials are reported, but we report both here for completeness. We consider this further in our discussion of Experiment 2 below.)

Overall, neither accuracy (Figure 3(a)) nor RT (Figure 3(b) and (c)) showed any evidence for viewpoint dependence in this experiment. As outlined in the *Introduction*, this was to be expected (e.g., White, 1980) because our letter rotation task can be performed using a feature identification strategy across the letter pairs and that does not require mental rotation.

The high-performance levels and similarity of results across groups are valuable demonstrations that both groups were engaged with our online task, and that any differences in our main experiment (Experiment 3) cannot be attributed to general differences in RTs or attentiveness across groups.

### Experiment 2: Mirror Reversals of Letters

#### Accuracy and RTs

The results for Experiment 2 are shown in the middle row of Figure 3. ANOVAs found highly significant main effects of orientation difference (viewpoint dependence) for accuracy (*p* < .001) (Figure 3(a)) and for RTs (Figure 3(b) and (c)) (*p* < .001) (Table A1 in the Appendix). This is consistent with previous work that also used mirror reversed letters (Cooper & Shepard, 1973; Corballis et al., 1978; Kail & Park, 1990; Koriat & Norman, 1985; Milivojevic et al., 2011; Núñez-Peña & Aznar-Casanonva, 2009; White, 1980) and suggests that our Experiment 2 successfully invoked mental rotation: as the orientation difference increased, more errors were made (Dalecki et al., 2012; Jolicoeur & Landau, 1984; Koriat & Norman, 1985; Smith & Dror, 2001) and RTs increased (Biederman & Gerhardstein, 1993; Shepard & Metzler, 1971). This strategy was to be expected because the simple feature identification strategy from Experiment 1 cannot be successful here since both letters in the pair contained the same features (Corballis et al., 1978).

#### Mental Rotation and Comparisons Across Groups

For the ‘same’ responses, the average increase in RT from an orientation difference of 0° to 180° was 1.36 s and 0.670 s for experts and novices, respectively, equivalent to mental rotation speeds of 132 and 269 deg/s (with the simplifying assumption of a linear relation between orientation difference and RT). Most studies of mental rotation for mirror reversed letters have used a single interval design where participants give either ‘normal’ or ‘mirror reversed’ responses on each trial. However, Kail and Park (1990) ran a design similar to our two spatial interval same/different design for mirror reversed letter-like stimuli (see Kail et al., 1980) and found an average rotation speed of 310 deg/s for adults. Using a similar approach, Mumaw et al. (1984) found a range of around 125–250 deg/s (their Figure 4). More generally, in a single interval standard mirror-letter task, Cooper and Shepard (1973) reported individual differences for adults ranging from 164 to 800 deg/s, and Takano (1989) ran both types of design and found similar results across the two. Against these conventional lab-based measures, our online results are within a normative range, if at the slow end for the expert group. However, the interaction between group and orientation difference (Table A1) was not significant for either accuracy (*p* = .511, BF_10_ = 0.58) or RT measures (*p* = .097, BF_10_ = 1.60), providing no support for group differences of mental rotation speeds.

**Figure 4.**
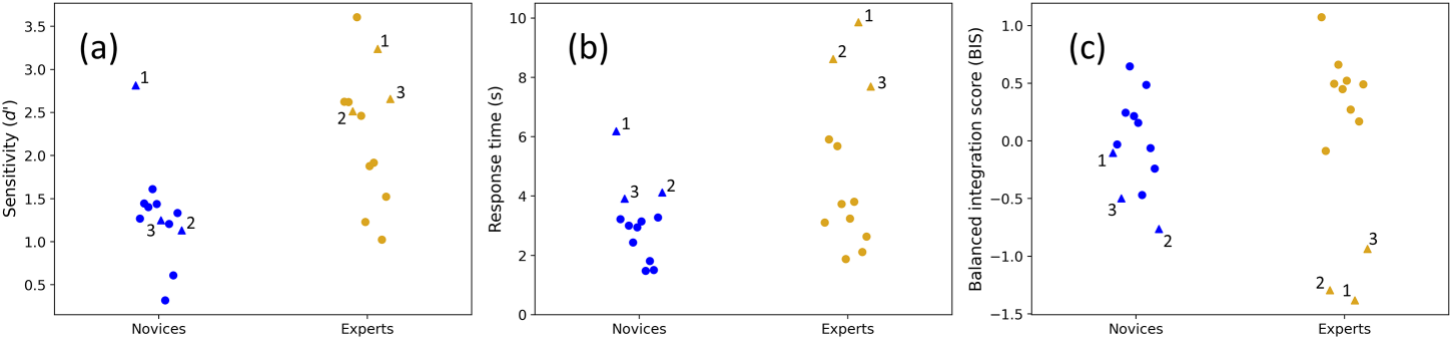
Group and participant comparisons for Experiment 3. Participant distributions are shown for (a) sensitivity, (b) RTs and (c) balanced integration scores (BIS), for ‘same’ and ‘different’ conditions combined, and averaged across orientation differences. Numbered triangles show the three slowest participants in each group. The mean and SDs for the novice group (blue) is 3.08 s (1.24 s) and for the expert group (gold) is 4.85 s (2.56 s). By excluding the three outlying experts (gold triangles), their means and SDs reduced to 3.56 s (1.34 s) – much closer to the 12 novices. For completeness, the mean and SDs of the nine fastest novices (blue circles), three slowest novices (blue triangles), and three slowest experts (gold triangles) were: 2.53 s (0.71 s), 4.74 s (1.02 s) and 8.72 s (0.89 s), respectively.

On the other hand, we did find group effects for both accuracy (*p* = .013) and overall RTs (*p* = .022) (Figure 3(d)–(f)), showing that the experts were more accurate but slower compared to the novices (Table A1 in the Appendix). Expert surveyors are trained to prioritize accuracy over speed, which might have contributed to this difference across groups. Furthermore, our novices were recruited from an online platform and had participated in many previous studies and surveys. Possibly then, they were more experienced in performing online experiments quickly and therefore prioritised speed over accuracy contributing further to this group difference.

We found a main RT effect for trial-type ('same’/'different’) (*p* = .03) and a highly significant interaction between trial-type and orientation (*p* < .001). (There were no other significant two-way or three-way interactions) (Table A1 in the Appendix). Evidently, the support for mental rotation derives largely from the ‘same’ and not ‘different’ letter pairs for both groups (compare the RT slopes in Figure 3(e) and (f)). This is consistent with Carter et al. (1983) who also found evidence for mental rotation in same/different comparisons for letter-like stimuli when they were the same, but less so when they were different. They explained this with a so-called deadline algorithm. On this model, participants respond as soon as they make a match across the stimulus pair, producing conventional RT functions for the ‘same’ condition (Figure 3(e)). However, in the ‘different’ condition they allow themselves extra time to succeed when the initial match fails (up to a self-imposed deadline), producing flat RT functions (Figure 3(f)). Minor deviations from flatness can be explained by supposing that the length of the deadline varies somewhat (Carter et al., 1983). Broadly speaking, this deadline idea is consistent with the observation that the longest RTs for the ‘same’ condition (right-hand side of Figure 3(e)) are similar to those in the ‘different’ condition (across Figure 3(f)) for each of the three functions. A similar observation can be made in Carter et al.'s (1983) Figure 4. Arguably, the influence of an external deadline is also evident in Smith and Dror's (2001) plots of error rate against orientation difference.

Overall, Experiment 2 showed that both groups engaged in a mental rotation-like strategy for our mirror reversed letter task.

### Experiment 3: Houses

Experiments 1 and 2 confirmed that participants (a) engaged with the general experimental requirements, (b) showed mental rotation-like behaviour in a 2D task where this was expected, but (iii) refrained from that strategy in a 2D task when a simpler viewpoint independent strategy was available. We turn now to our main experiment (Experiment 3) where we asked whether either group of participants employed a mental rotation strategy for the 3D task of recognising a house across ground and aerial views, whether experts were more adept at this task than novices and whether any other differences across groups might emerge.

#### Accuracy

The results for Experiment 3 are shown in the bottom row of Figure 3. Experts were more accurate (Figure 3(g); Table A1 in the Appendix) than novices (average accuracy was 86.2% [*d*’ = 2.27] and 73.4% [*d*’ = 1.32] for experts and novices, respectively). However, a numerical comparison with the mirror letter task showed that the expert facility in the house task was not specific to aerial views: average accuracies for the mirror letters in Experiment 2 were *d*’ = 2.18 and *d*’ = 1.18 for experts and novices, respectively (Figure 3(d)).

ANOVA found a main effect of orientation difference for accuracy (*p* = .004; BF_10_ = 7.72; Table A1 in the Appendix). However, this was not the classic decreasing function with orientation difference that is the signature of mental rotation (Dalecki et al, 2012; Frick et al., 2013; Hamm et al., 2004; Jost & Jansen, 2022; Klotzbier & Schott, 2024; Koriat & Norman, 1985; Milivojevic et al., 2011; Smith & Dror, 2001; Voyer et al., 2017), but a gentle U-shaped function.

We consider the result above uninteresting for two reasons. First, the effect size is very small, accounting for less than 5% of the variance (η^2^_G_ = 0.049). Where accuracy has been plotted in mental rotation studies (e.g., Smith & Dror, 2001), its effect is much larger than this (by eye) when mental rotation is found. Second, if this result were a genuine indicator of mental rotation, then RTs should also show the same effect (e.g., Dalecki et al., 2012; Koriat & Norman, 1985; Milivojevic et al., 2011) – they do not (Figure 3(h); see also below).

For these reasons, we do not pursue an explanation in terms of nonlinear factors (in the Shepard & Metzler (1971) sense) based on results for the naming of familiar objects against orientation (Lawson & Jolicoeur, 2003). In fact, such nonlinearity (convex upwards) is not seen in conventional 3D mental rotation tasks (Jolicoeur et al., 1985, 1998), and familiar stimuli in a conventional mental rotation task produce curvilinear trends in the opposite direction (convex downwards) to those in Lawson and Jolicoeur (Koriat & Norman, 1985).

In sum, it remains unclear what processes lay behind our small statistical effect for accuracy reported above but, in principle, it could derive from detail image differences across orientation.

#### Bias

We calculated a bias measure for each observer by subtracting the number of ‘different’ responses from the number of ‘same’ responses and normalising by the total. We found a very small bias towards ‘different’ responses (−0.025 and −0.030 for experts and novices, respectively) but there was no significant difference across groups [*t*(22) = 0.497, *p* = .624]. We repeated this analysis using the bias measure (*c*) from SDT but this did not change our conclusions [*F*(1, 22) = 1.02, *p* = .324, η^2^_G_ = 0.029]. And neither did plotting this measure against orientation difference [*F*(4, 88) = 0.396, *p* = 0.811, η^2^_G_ = 0.006]. We do not consider these bias measures further.

#### RTs

There was no main effect of orientation difference for RTs (*p* = .3). This was confirmed by the Bayes factor (BF_10_ = 0.108) providing evidence against mental rotation for this experiment. However, the ANOVA for Experiment 3 (Tables A1 in the Appendix) revealed a significant interaction between orientation and trial-type (*p* = .01). This is barely evident by eye in Figure 3(h) and (i), and was not supported by BF_10_ = 0.94. Nonetheless, this prompted us to remove trial-type as a factor and perform a two-way ANOVA on the RTs for the ‘same’ condition alone. This confirmed there was no main effect for orientation difference.

Overall, we conclude that mental rotation took place for letters in Experiment 2, but that aerial images were not mentally rotated when matching houses across ground and aerial viewpoints in Experiment 3. We suggest that a form of feature identification strategy might have been used instead and provide a deeper consideration of this point in the General discussion*.*

#### Overall Speed and Individual Differences Within the Expert Group

Average expert and novice RTs for houses were, 4.85 s and 3.08 s, respectively (Figure 3(h) and (i)), which were significantly slower than the corresponding RTs of 2.07 s and 1.42 s for the mirror letter task (Figure 3(e) and (f)) [*F*(1, 44) = 24.39, *p* < .001, η_p_^2^ = 0.357]. Unlike in Experiment 2, the speed difference across groups did not reach statistical significance for the houses (*p* = .051; Table A1 in the Appendix) but there was no interaction between group and experiment for RTs (*p* = .196) and the ANOVA conducted on the ‘same’ condition alone (reported above) did find a significant effect of group [*F*(1, 22) = 4.36; *p* = .049, η_p_^2^ = 0.165].

This marginal group effect prompted us to examine the speed results more closely by looking at individual participants within each group. A full comparison of RTs is shown in Figure 4(b) where the implication is self-evident. The distribution of the 12 novice RTs (blue symbols) is similar to the nine fastest experts (gold circles) (means and SDs of 3.08 s(1.24 s) and 3.56 s(1.34 s), respectively). The three slow outliers are shown by the gold triangles, illustrating the greater diversity of RTs amongst the experts than across the novices. It follows that the difference across groups in Experiment 3 (Figure 3, bottom row) is carried largely by just 25% (three out of 12) of the participants in the expert group who are notably slower than the other participants.

**Figure 5.**
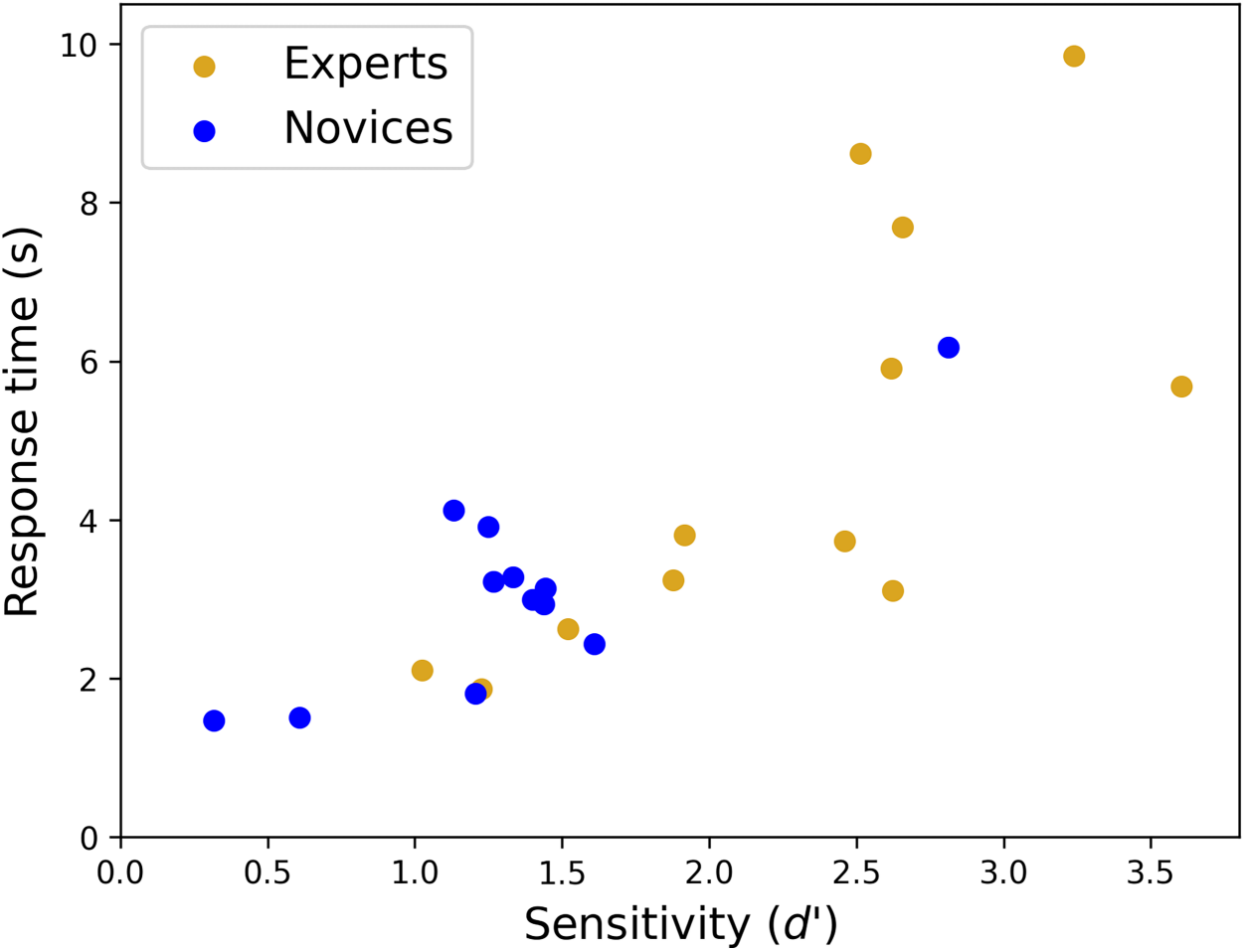
Response times verses sensitivity for the experts (gold symbols) and novices (blue symbols) in Experiment 3.

The distributions of sensitivities are shown in Figure 4(a). While the three slowest experts (gold triangles) tend towards the upper part of the distribution, they are not the three most accurate participants. Nonetheless, the square of Pearson's correlations for RT against sensitivity (collapsed across group) for the novices, experts, and combination of both groups, was *r*^2^ = 0.68 (df = 10; *p* < .001), 0.53 (df = 10; *p* = .007) and 0.62 (df = 22; *p* < .001), respectively. This is consistent with speed-accuracy trade-offs within and across groups (see Figure 5). The unaccounted for variance presumably includes a component of indecision in this visuo-cognitively challenging task. In fact, we found that the pooled within-observer SD for the slow experts (gold triangles) was remarkably high (4.66 s) consistent with a trial dependent strategy where correctness is prized and the decision time taken is that which is needed to make the judgement with confidence.

Overall, we conclude that the experts were more accurate than novices in the house experiment, but the group differences in RTs (Figure 3(h)) arose because some experts (*n* ∼ 3) took longer to make their decisions than others (Figures 3 and 4).

Speed-accuracy trade-offs for RT tasks are well-known in the literature and subject to external influences such as whether instructions emphasise speed or accuracy (e.g., Hertzog et al., 1993). However, since the instructions were identical for all participants in our experiments, the implication is that different observers brought different emphases with them to the task. Judging by the visual segmentation of novices and experts in Figure 5, and the significant differences across group for accuracy and RTs, we conclude that these different emphases were different across groups.

Finally, we wondered whether a difference across groups might be found after allowing for speed-accuracy trade-offs in the analysis. To do this, we computed balanced integration scores (BIS) where percent correct scores and RTs were converted to *Z*-scores,^3^ and the latter subtracted from the former (Liesefeld & Janczyk, 2019). For example, it could be that overall, the higher accuracy amongst the experts betters that expected by their longer RTs. However, a *t*-test across groups found no significant effect for BIS [*t*(22) = 0.269, *p* = .791], as might be anticipated by eye from Figure 4(c).

For completeness, we also performed the BIS analysis for Experiment 2 and found no significant effect [*t*(22) = 0.312, *p* = .758], consistent with our assumption of a speed-accuracy trade-off, as in Experiment 3; the correlation was lower but significant [*r*^2^(22) = 0.23, *p* = .048].

## General Discussion

### Study Overview

We investigated group differences between novices from the general population and expert remote sensing surveyors from the OS in a task of object matching (same/different) of houses across ground and non-canonical aerial views. The viewpoints being compared varied substantially around two orthogonal spatial axes so that if mental rotations were to take place, they would operate through a three-dimensional space. We considered this to be a suitably challenging task for our workplace trained observers (Skog et al., 2024, 2025) where visual expertise might show itself. Our line of enquiry involved two primary issues: (1) the visuo-cognitive strategies involved in relating aerial and ground view images of objects, and (2) the potential benefits of experience for performing this task. Two ‘grounding’ experiments tested the participants on basic 2D letter tasks and established that both participant groups produced results consistent with a feature identification strategy (Experiment 1) and a mental rotation strategy (Experiment 2) as appropriate for the visual information available. In our main (house) experiment (Experiment 3) we found that responses were much slower than in the two letter tasks, but that neither participant group was constrained by mental rotation time when same/different judgments of houses were made across viewpoints. We also found a benefit of experience with aerial images, where the experts made more accurate identifications of houses across 3D changes in viewpoint. However, a similar effect was found in the letter rotation experiment where mental rotation was required, suggesting that the expert benefit was not specific to aerial images but greater diligence in our same/different tasks when they became taxing and ceiling effects absented (Experiments 2 and 3). (By diligence, we mean driven for accuracy and taking the time to do this if necessary.) For some of the expert observers, this came at the cost of slower RTs by comparison to most of the novices (i.e., an accuracy speed trade-off), but not for them all (see Figure 5).

While our experiments fall into the general category of spatio-cognitive decision making and a literature borne from the early 70s (Shepard & Metzler, 1971), they also relate to much later developments in the broader context of object recognition. We contextualise our results in each of these movements in what follows before turning to visual expertise.

### Object Recognition: The Well-Trodden Path

Generally speaking, there are two possible routes to object recognition: object-centred (Biederman, 1987; Marr & Nishihara, 1978) and viewpoint dependent (Edelman & Bülthoff, 1992; Newell & Findlay, 1997; Tarr et al., 1998). Following a rich debate (e.g., Biederman & Gerhardstein, 1993, 1995; Biederman & Bar, 1999; Bülthoff et al., 1995; Tarr & Bülthoff, 1995, 1998; Hayward & Tarr, 2000; Lawson, 1999) the consensus is that human vision involves both (Hayward, 2003; Hummel, 2000) subject to stimulus constraints and task demands (Biederman & Gerhardstein, 1993; Blais et al., 2009; Lawson, 1999; Rentschler et al., 2008; Tarr & Hayward, 2017) with elegant modelling solutions for combining the two into a single decision variable (e.g., Foster & Gilson, 2002). More recent work involving deep neural nets has delivered remarkable performance for object recognition in machine vision, but how these processes relate to human vision remains to be understood (Heinke et al., 2022; Leek et al., 2022). Our house experiment was not designed to contribute to any of these debates, but we can consider our results alongside them. One cost of viewpoint dependent representations is an increase in error rates (Edelman & Bülthoff, 1992) and RTs (Tarr & Pinker, 1989) with orientation difference across viewpoints (Takano, 1989), whereas object-centred representations are viewpoint invariant and indifferent to object orientation (within certain constraints) (Biederman & Gerhardstein, 1993). At first glance, this puts our house results in the object-centred camp, but the long RTs for that experiment prompt further consideration as follows.

### Task Strategy for Our Houses (Experiment 3)

Studies that typically find strong effects of mental rotation commonly involve judgements of rotated abstract shapes, such as in the traditional brick figures of Shepard and Metzler (1971) where local features are not diagnostic but object configuration is. In other words, specific tasks call for a mental rotation strategy, as in our mirror reversed letter task (Experiment 2), but what might take place for our houses?

We note that the task here was more complex than typical mental rotation experiments because some features were not visible in both images (e.g., a full view of the roof was visible only from the air; see also, Just & Carpenter, 1985) and presumably these accretions and deletions of features with viewpoint added to the overall RTs (Biederman & Gerhardstein, 1993).

On this matter, first we note a twins study (Shakeshaft et al., 2016) involving a genetic component for spatial ability. The authors found that the mental visualisations needed to deal with the complex tasks involving missing features did not dissociate from mental rotation skills. In other words, we might expect that those who can mentally rotate are also well disposed to dealing with the accretion and deletion of image features in viewpoint invariant object recognition, such as those in our houses task.

Second, and more importantly, although our task was probably more demanding than conventional spatio-cognitive tasks, that in itself does not rule out a potential role for mental rotation. For example, a strategy involving repeated attempts at mental rotation to overcome the visuo-cognitive demands of complex feature sets (Just & Carpenter, 1985; Yuille & Steiger, 1982) would show an effect of orientation difference on RT, nonetheless. Indeed, it has been suggested that conventional mental rotation is in fact piecemeal (Larsen, 2014; Yuille & Steiger, 1982) even for Shepard and Metzler stimuli when presented in 2D (Stark et al., 2024). Another relevant factor is that for most studies that find mental rotation, the ‘different’ stimulus is defined by a mirror reversal (Corballis & McLaren, 1984) whereas here, the unmatched comparison stimuli were photographs of different houses. However, on developing the Shepard and Metzler stimuli, Yuille and Steiger (1982) found long RTs (comparable with those here) for particularly complex ‘same’ stimuli and strong evidence for mental rotation when the ‘different’ stimuli were defined by twisted segments rather than an overall mirror reversal (rotation speeds were as slow as 33 deg/s). Similarly, Just and Carpenter (1985) found long RTs and evidence for mental rotation using pictorial cubes where ‘different’ stimuli were defined by feature manipulations on one or two of the faces. Thus, an experimental design based on mirror reversing is not a pre-requisite for finding mental rotation. Of course, it remains possible that we might have found evidence for mental rotation for houses if the task had been designed for detecting mirror reversals and this remains a possibility for future work. So then, what type of visual strategy might our participants have been using in our house experiment?

Three strategies have been identified for spatio-cognitive comparison tasks (Schultz, 1991). These are the mental rotation and feature identification strategies outlined in the Introduction, but another possibility is that rather than rotating the houses, participants rotated their viewpoints (Just & Carpenter, 1985; Koriat & Norman, 1984), sometimes known as spatial perspective taking (Hegarty & Waller, 2004; Zacks et al., 2002a, 2002b). Although these two types of rotation are formally equivalent in terms of image data transforms for the observer, they have been dissociated behaviourally (Hegarty & Waller, 2004; Simons & Wang, 1998), have different neural signatures (Keehner et al., 2006; Kosslyn et al., 1998; Zacks & Michelon, 2005;), different incidences according to task (see Just & Carpenter, 1985; Schultz, 1991) and have different dependencies on orientation difference, with viewer rotations approaching invariance according to errors and RTs (Amorim & Stucchi, 1997; Dalecki, et al., 2012; Voyer et al., 2017; Wraga et al., 2000; Zacks et al., 2002a, 2002b). It has been suggested that this last result obtains because in mental rotation, the representation of the object passes along a time-consuming smooth trajectory, whereas in perspective taking, the observer ‘jumps’ to the desired viewpoint directly (Wraga et al., 2000), sometimes called a blink transform (see Wraga et al., 2000; Zacks & Michelon, 2005). While conventional mental rotation is a natural strategy for objects that are susceptible to manhandling (e.g., a kettle or a chair), it seems far less natural to do this for an entire scene or large, rooted objects such as houses, where an egocentric reference frame is more appropriate (e.g., see Dalecki et al., 2012). This makes a perspective transform seem like a more natural choice than mental rotation of the object in our house experiment or a distinct possibility, at least. Furthermore, perspective-taking rotations have been associated with viewpoint-independent recognition (Simons & Wang, 1998).

When tasks are demanding (e.g., owing to the complexity of the stimuli), as demonstrated by higher errors rates (e.g., Figure 3(g)), then participants might search for a strategy other than rotation, such as feature identification (Yuille & Steiger, 1982). If there are sufficiently tell-tale features clear in both images – e.g., a giant fish sculpture sticking out of the roof (“Headington Shark”, n.d.) or other unique abstract features (Eley, 1982) – then the critical information is not orientation-bound (Eley, 1982; Takano, 1989) and comparisons can be made without an object-centred representation. While this is almost certainly the basis for behaviour in Experiment 1, we think that in its basic form this strategy is unlikely in Experiment 3 for two reasons. First, we screened our stimuli for roof sharks and other immediate giveaways. Second, if such giveaways were evident in our stimuli, we would expect short RTs (see Experiment 2 in Takano, 1989) not the long ones we found. A related possibility is that tell-tale features were not self-evident (i.e., did not ‘pop out’, like a roof shark), but required participants to search the two images to find and compare them. This is a reasonable description of our own experiences with these stimuli, but the strategy requires cross-registration between the features under scrutiny and their positions on the objects. This is an inherently object-centred component of the task that at the very least requires a perceptual grasp of the spatial relations between visible stimulus surfaces (Leek et al., 2005; Reppa et al., 2015). In sum, we think our house task might plausibly tap a (geon-like) three-dimensional volumetric prototype model of a house (Biederman, 1987; Biederman & Bar, 1999) for the basis of feature/component comparisons across viewpoints and surfaces, but a connected 3D description of visible surfaces (Arguin et al., 2019; Leek et al., 2005) would probably suffice. Either way, a perspective taking strategy by our participants would presumably also help.

### Group Differences Across Experts and Novices

Previous work has found benefits of training (Borders et al., 2020; Lloyd et al., 2002) and expertise for aerial image tasks. Lansdale et al. (2010) showed that for urban and rural scenes, OS remote sensing surveyors (*n* = 7) were marginally better than the same number of novices in a change detection task and clearly better in a location memory task. From eye-movement results, experts were also better at overriding the draw of salient image features. In a recognition task involving five-second learning for each of 72 (urban) aerial images, Šikl et al. (2019) compared expert remote sensing surveyors (*n* = 39) with similarly sized groups of psychology and geography students. Geography students had some anecdotal experience with aerial images, but neither student group rivalled the performance of the experts in forced-choice recognition of the target images against other aerial distractors. This was interpreted as an expert advantage in associating semantic information with visual information (i.e., a strategy benefit). However, like in our own work, the performance benefit for the expert group came at the cost of slower RTs suggesting a speed-accuracy trade-off in favour of accuracy. Furthermore, performance for all of Šikl et al.'s participants declined when the correct comparison image was rotated by 90° (in the image plane) indicating a lack of viewpoint invariance for their task. However, Šikl et al.'s task tapped one-shot memories of previously viewed aerial scenes, whereas our task was very different, involving simultaneous spatial comparisons of objects (houses).

In our previous work on CIs for aerially viewed hedges and ditches, Skog et al. (2024) showed a large OS expert advantage over novices for extracting useful signal from binocular disparity. This was a very different perceptual strategy from the novice observers whose depth judgements placed greater emphasis on lighting and shading. Thus, for aerial images, Skog et al. (2024), Lansdale et al. (2010) and Šikl et al. (2019) all found evidence for expert strategy. However, the differences between aerial experts and novices does not always lie in strategy. In a study on gist categorisation for briefly presented aerial- and ground-view images, Skog et al. (2025) found differences in degree rather than kind across OS experts and novices.

The results of the present study are rather more like those of Skog et al. (2025) than the others summarised above, in that only diligence not strategy appeared to differ across participant groups. While our OS experts were undoubtedly more familiar with aerial viewpoints of houses than our novices, we suggest that it was the experts’ transferable skill of care and attention (Šikl et al., 2019; Skog et al., 2025) that showed up as the primary cause of group differences here (for houses and mirror-reversed letters), rather than specific visuo-cognitive expertise. Of course, we cannot know the inner workings of participants in either group, only their data. The differences across the two groups are clear but we cannot rule out the possibility that factors other than experience from training might be involved. For example, it could be that the group differences derive from the scrutiny involved in partaking in an experiment, and that this was more pressing for the experts.

## Conclusions

Mental rotation plays no apparent role in a same/different object recognition task across aerial and ground-based viewpoints of houses for experts or novices. Instead, we suggest that participants performed our main task by identifying features across the images in a viewpoint invariant framework, though rapid perspective shifting might have also taken place. Experience with aerial images had no obvious benefit for the specifics of our task involving aerial views of houses. However, more generally (houses and letters), experts were more accurate than novices (excluding the first experiment for which there was a ceiling effect) – presumably a product of care from their training. Indeed, we also found this general diligence amongst experts in a novel (for them) mirror reversed letter rotation task.

## Notes

1. Many of the houses were conjoined pairs of semi-detached homes that appear as one building. Here, we also refer to such structures as houses.

2. Some participants had a hit proportion of 1.0 and a false alarm proportion of 0.0 which produces an infinitely high *d*’ estimate of sensitivity. This mainly occurred in Experiment 1 (and is an inherent problem with single-interval binary choice tasks such as the one here). To circumvent the problem of infinite *d*’ measures in averaging, we applied the log-linear rule for correction to all measures of *d*’ as described by Hautus (1995).

3. The analysis was done for Experiment 3 (houses) collapsing across the ‘same’ and ‘different’ results and deriving the sample standard deviation across groups at each orientation difference to produce a BIS for each participant at each orientation difference. For each participant, their BIS was then averaged across orientation difference.

## Appendix

**Table A1.** Results of Statistical Analyses (ANOVA and BF) Reported in the Main Body Text.

	Expt 1	Expt 1	Expt 2	Expt 2	Expt 3	Expt 3
Dependent variable	Letter rotation accuracy (*d*’)^a^	Letter rotation RT^a^	Mirror reversal accuracy (*d*’)^a^	Mirror reversal RT ^a,b^	House accuracy (*d*’)	House RT^a^
*Main effects*	** **					
Orientation	*F*(2.61, 57.42) = 2.96, *p* = .023*, η^2^_G_ = 0.089, BF_10_ = 1.90	*F*(1.45, 31.85) = 2.32, *p* = .128, η^2^_G_ = 0.015, BF_10_ = 0.561	*F*(3.04, 66.83) = 5.77, *p* < .001***, η^2^_G_ = 0.072, BF_10_ = 55.133	*F*(2.53, 55.65) = 16.97, *p* < .001***, η^2^_G_ = 0.092, BF_10_ = 6.730 × 10^12^	*F*(4, 88) = 4.16, *p* = .004**, η^2^_G_ = 0.049, BF_10_ = 7.721	*F*(3.00, 65.97) = 1.245, *p* = .301, η^2^_G_ = 0.002, BF_10_ = 0.108
Group	*F*(1, 22) = 0.69, *p* = .416, η^2^_G_ = 0.010, BF_10_ = 0.248	*F*(1, 22) = 0.46, *p* = .503, η^2^_G_ = 0.016, BF_10_ = 0.307	*F*(1, 22) = 7.28, *p* = .013*, η^2^_G_ = 0.189, BF_10_ = 3.313	*F*(1, 22) = 6.05, *p* = .022*, η^2^_G_ = 0.150, BF_10_ = 3.263	*F*(1, 22) = 11.4, *p* = .003**, η^2^_G_ = 0.273, BF_10_ = 9.272	*F*(1, 22) = 4.26, *p* = .051, η^2^_G_ = 0.142, BF_10_ = 0.547
Trial–type	*–*	*F*(1, 22) = 0.10, *p* = .753, η^2^_G_ = 0.000, BF_10_ = 0.248	–	*F*(1, 22) = 5.42, *p* = .030*, η^2^_G_ = 0.033, BF_10_ = 5.700 × 10^6^	–	*F*(1, 22) = 0.44, *p* = .513, η^2^_G_ = 0.002, BF_10_ = 0.369
*Two–way*						
Orientation × Group	*F*(2.61, 57.42) = 0.74, *p* = .517, η^2^_G_ = 0.014, BF_10_ = 0.088	*F*(1.45, 31.85) = 0.34, *p* = .645, η^2^_G_ = 0.002, BF_10_ = 0.101	*F*(3.04, 66.83) = 0.83, *p* = .511, η^2^_G_ = 0.011, BF_10_ = 0.583	*F*(2.53, 55.65) = 2.3, *p* = .097, η^2^_G_ = 0.014, BF_10_ = 1.596	*F*(4, 88) = 0.14, *p* = .968, η^2^_G_ = 0.001, BF_10_ = 0.258	*F*(3, 65.97) = 0.89, *p* = .450, η^2^_G_ = 0.001, BF_10_ = 0.028
Trial–type × Group.	*–*	*F*(1, 22) = 1.89, *p* = .183, η^2^_G_ = 0.004, BF_10_ = 0.227	–	*F*(1, 22) = 1.76, *p* = .199, η^2^_G_ = 0.011, BF_10_ = 1.543	–	*F*(1, 22) = 0.95, *p* = .340, η^2^_G_ = 0.004, BF_10_ = 0.497
Orientation × Trial–type	*–*	*F*(2.68, 59) = 3.00, *p* = .043*, η^2^_G_ = 0.008, BF_10_ = 0.611	–	*F*(2.78, 61.22) = 13.09, *p* < .001***, η^2^_G_ = 0.050, BF_10_ = 7.851 × 10^6^	–	*F*(2.85, 62.76) = 3.13, *p* = .034*, η^2^_G_ = 0.005, BF_10_ = 0.335
*Three–way*						
Orientation × Group × Trial–type	–	*F*(2.68, 59) = 0.32, *p* = .787, η^2^_G_ = 0.001, BF_10_ = 0.008	–	*F*(2.78, 61.22) = 0.78, *p* = .501, η^2^_G_ = 0.003, BF_10_ = 0.576	–	*F*(2.85, 62.76) = 0.51, *p* = .669, η^2^_G_ = 0.001, BF_10_ = 0.001

*Note.* Bayes factors (BF) and results of mixed repeated measures ANOVAs for accuracy (*d*’) and response times (RT) in all three experiments. The factors (left column) are ‘Orientation’ difference (0–180°), ‘Group’ (expert, novice) and ‘Trial-type’ ('same’ or ‘different’). Significant cells (alpha = 0.05) are indicated thus: **p* < .05, ***p* < .01, ****p* < .001. × = interactions.

a Greenhouse-Geisser corrected column following a positive sphericity test.

b Analyses of RTs were always based on correct responses, but in Experiment 2, some participants (one expert and five novices) provided no correct responses in at least one image category (e.g., ‘same’ image, 45° orientation difference). Such image categories consisted of six trials each. To avoid excluding participants on this basis, 13 out of 240 cells were filled with the average of the other available participants in the same group and condition.
